# Supplementary material for: Mixed hierarchical local structure in a disordered metal–organic framework
Source: Nat Commun. 2021 Apr 6;12:2062. doi: 10.1038/s41467-021-22218-9 (PMC8024318; doi:10.1038/s41467-021-22218-9)
Supplement: Supplementary file 1 — Supplementary Information [file 41467_2021_22218_MOESM1_ESM.pdf]

Mixed Hierarchical Local Structure in a Disordered Metal–Organic Framework

SUPPLEMENTARY INFORMATION

Adam F. Sapnik[1] Irene Bechis,[2] Sean M. Collins,[1,3] Duncan N. Johnstone,[1] Giorgio Divitini,[1] Andrew J. Smith,[4] Philip A. Chater,[4] Matthew Addicoat,[5] Timothy Johnson,[6] David. A. Keen,[7] Kim E. Jelfs,[2] Thomas D. Bennett[1]*

1 Department of Materials Science and Metallurgy, University of Cambridge, Cambridge, CB3 0FS, UK.

2 Department of Chemistry, Imperial College London, Molecular Sciences Research Hub, White City Campus, London, W12 0BZ, UK.

3 School of Chemical and Process Engineering & School of Chemistry, University of Leeds, Leeds, LS2 9JT, UK.

4 Diamond Light Source Ltd, Diamond House, Harwell Campus, Didcot, Oxfordshire, OX11 0DE, UK.

5 School of Science and Technology, Nottingham Trent University, Clifton Lane, Nottingham, NG11 8NS, UK.

6 Johnson Matthey Technology Centre, Blount's Court, Sonning Common, Reading, RG4 9NH, UK.

7 ISIS Neutron and Muon Facility, Rutherford Appleton Laboratory, Harwell Campus, Didcot, Oxfordshire, OX11 0QX, UK.

* To whom correspondence should be addressed; E-mail: tdb35@cam.ac.uk

Supplementary Figures

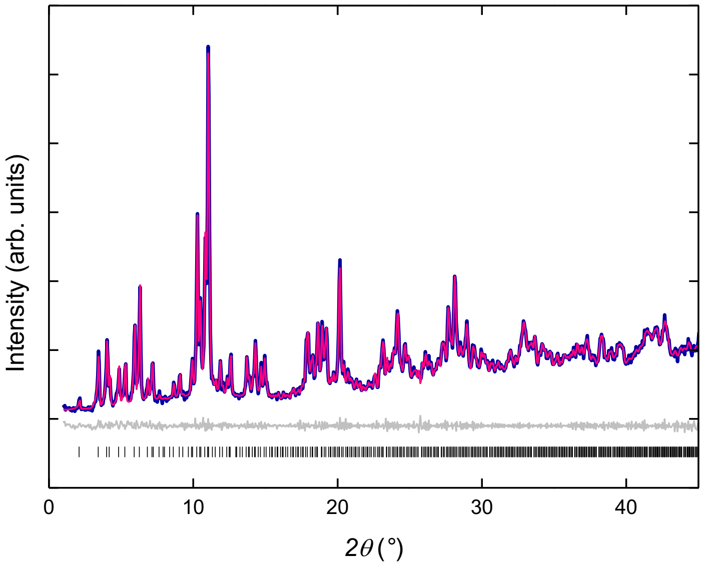

Supplementary Figure 1 Pawley refinement of MIL-100 (Fe). Experimental data (blue), calculated diffraction pattern (pink), difference function (grey) and symmetry-allowed reflections (black ticks). Symmetry-allowed reflections were calculated from the reported crystallographic information file (CCDC identifier: 640536).

Supplementary Table 1 Crystallographic data from Pawley refinement of MIL-100.

R_{wp} = 5.78	Experimental	Calculated
a = *b* = *c*	73.23(1)	73.340(1)
α = β = γ	90	90

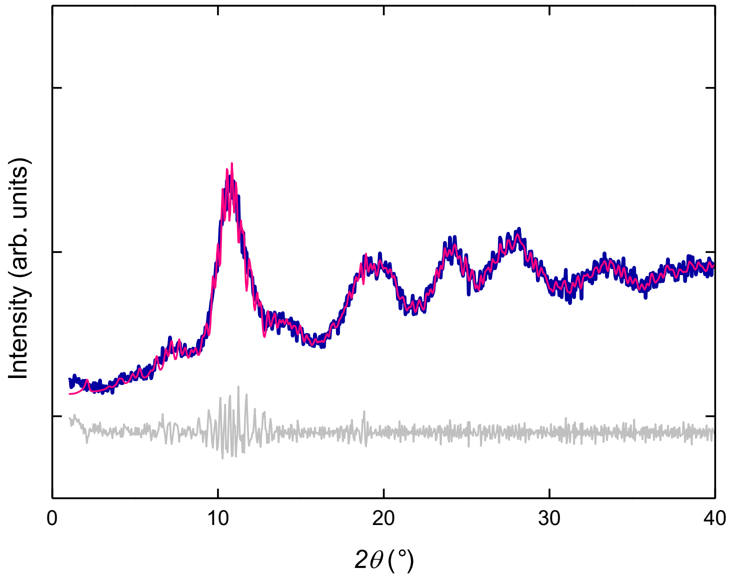

Supplementary Figure 2 Pawley refinement of Fe-BTC powder X-ray diffraction data. Experimental data (blue) calculated data using a crystallite size constrained to 40 nm (pink) and difference function (grey).

Supplementary Table 2 Crystallographic data from Pawley refinement of Fe-BTC. It is important to note that little physical significance is given to this refinement as the peaks are much broader than the typical separation of MIL-100's Bragg peak positions and it is simply illustrating that such diffraction data can lead to successful convergence of a refinement when considering such small domain sizes.

R_{wp} = 7.50	Experimental
$a = b = c$	73.24(4)
$\alpha = \beta = \gamma$	90

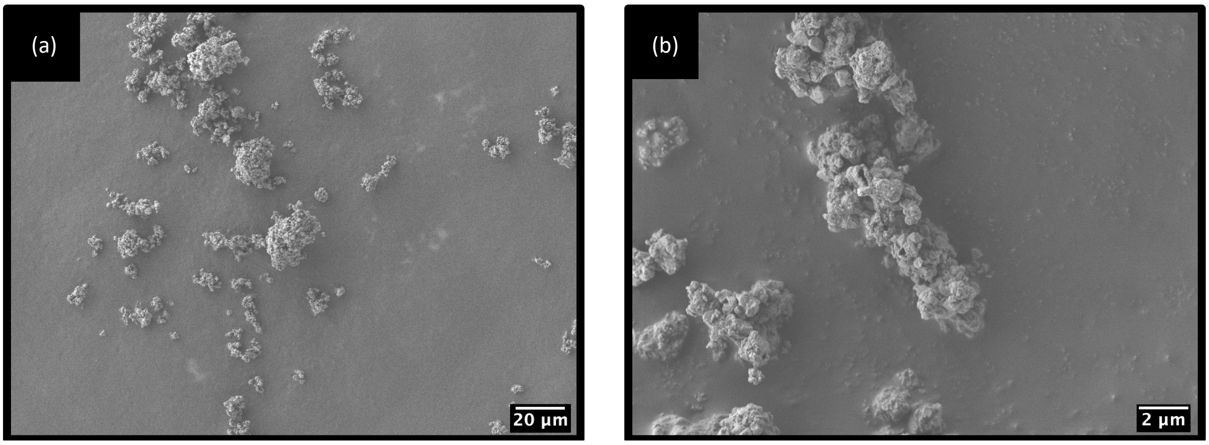

Supplementary Figure 3 Scanning electron micrographs of MIL-100 (a-b).

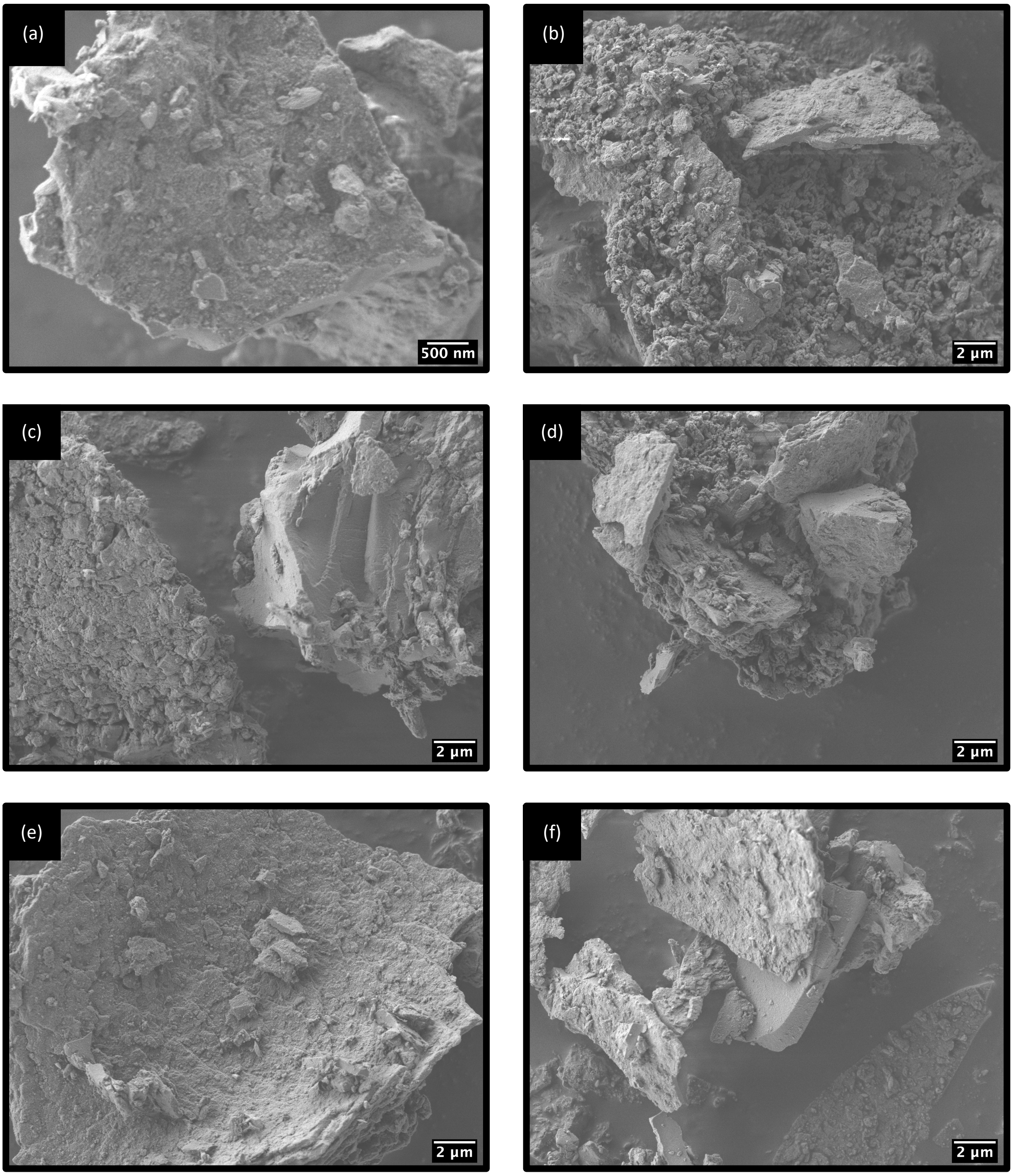

Supplementary Figure 4 Scanning electron micrographs of Fe-BTC (a-l).

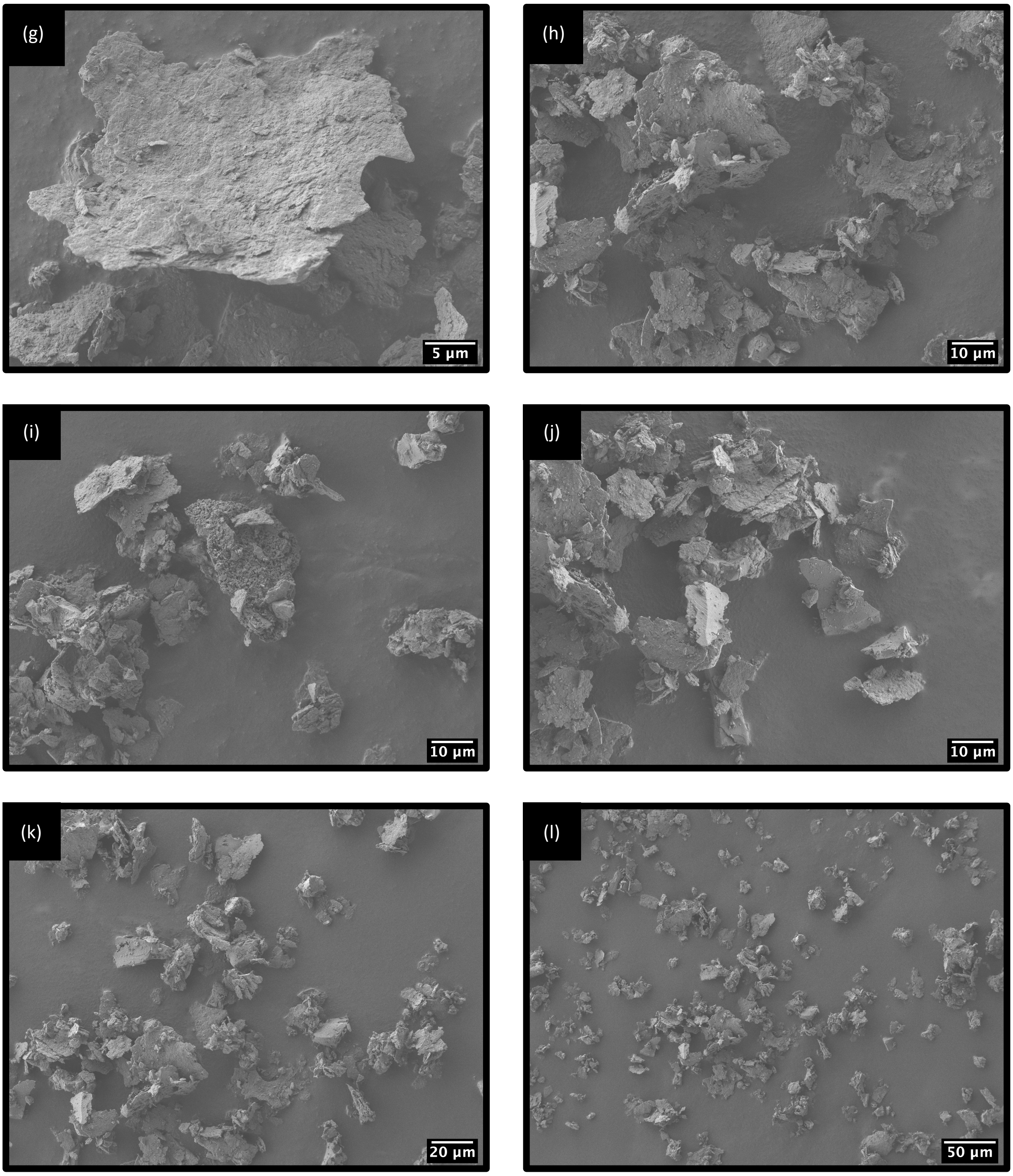

Supplementary Figure 4 Scanning electron micrographs of Fe-BTC (a-l).

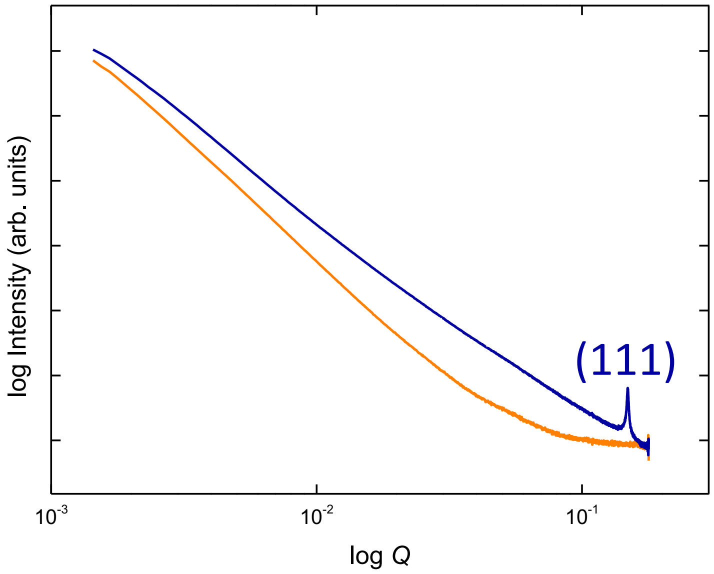

Supplementary Figure 5 Logarithmic plot of small-angle X-ray scattering data from MIL-100 (blue) and Fe-BTC (orange).

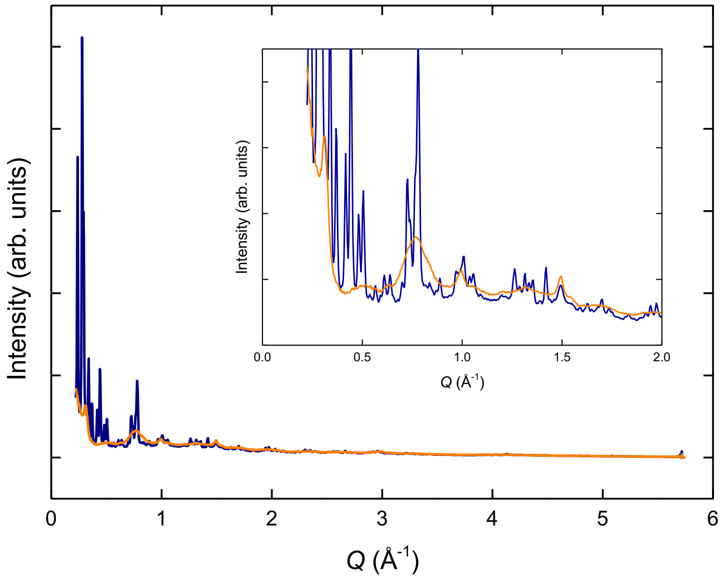

Supplementary Figure 6 Wide-angle X-ray scattering data from MIL-100 (blue) and Fe-BTC (orange). Inset shows the low-*Q* region

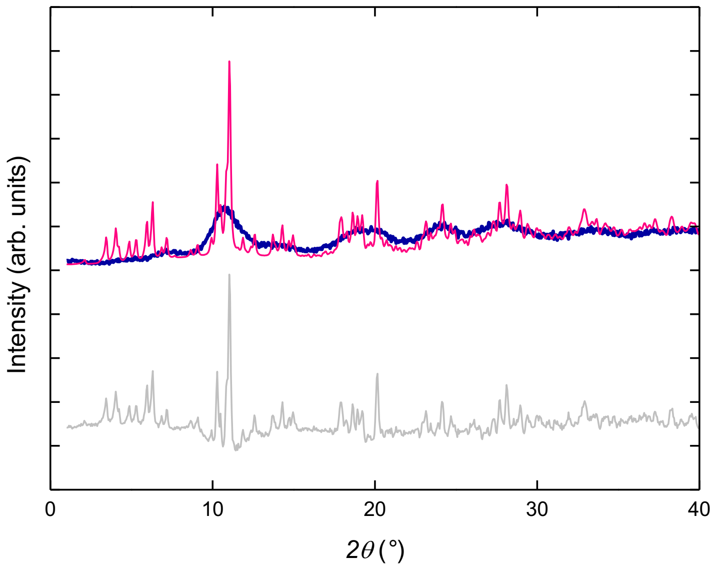

Supplementary Figure 7 Simulated nanocrystalline diffraction pattern of MIL-100 using a crystallite size constrained to 200 nm (pink), experimental Fe-BTC diffraction pattern (blue) and difference function (grey).

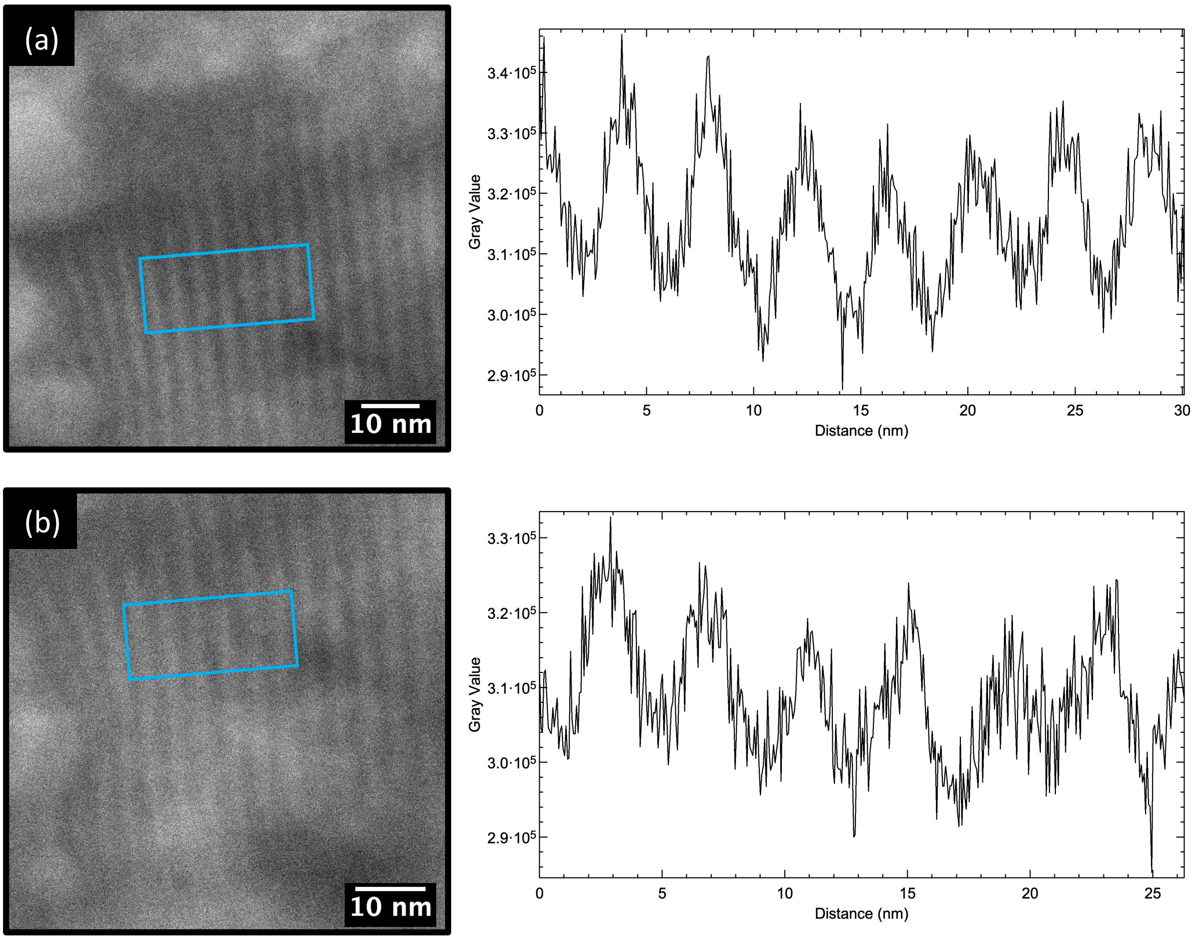

Supplementary Figure 8 Bright field (a) and high-angle annular dark-field (b) HR-STEM images of MIL-100 and their corresponding line profile measurements of the highlighted lattice fringes. Distances between minima are approximately 4.2 nm and correspond to the *d* spacing between (111) crystallographic planes.

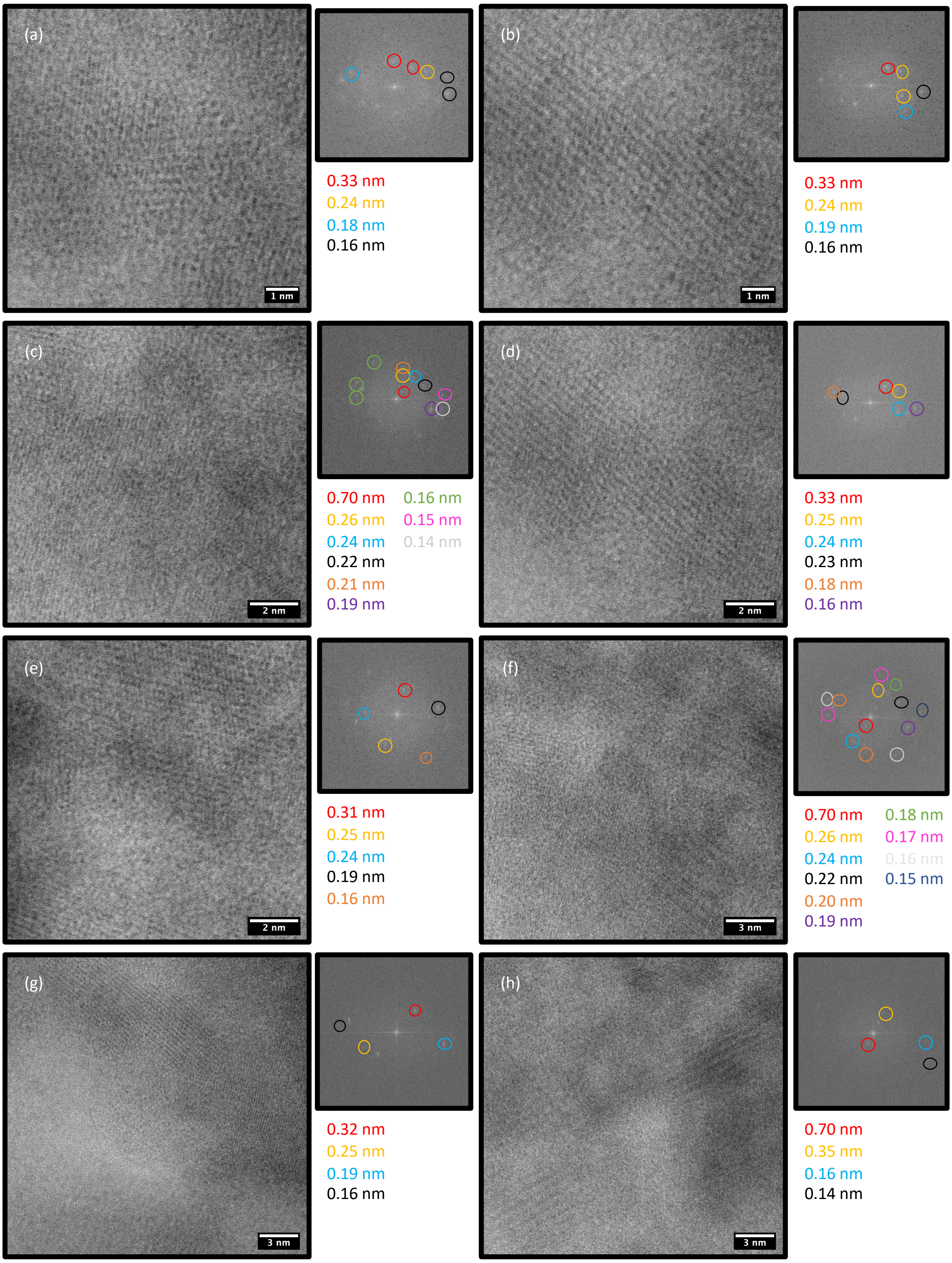

Supplementary Figure 9 Bright field (a-n) and high-angle annular dark field (o-p) HR-STEM images of Fe-BTC. Insets show the Fourier transform of the corresponding micrograph; reciprocal space features correspond to periodic features in the real space image. Coloured circles are used to highlight reciprocal space features and colour-coded distances are given below the Fourier transform.

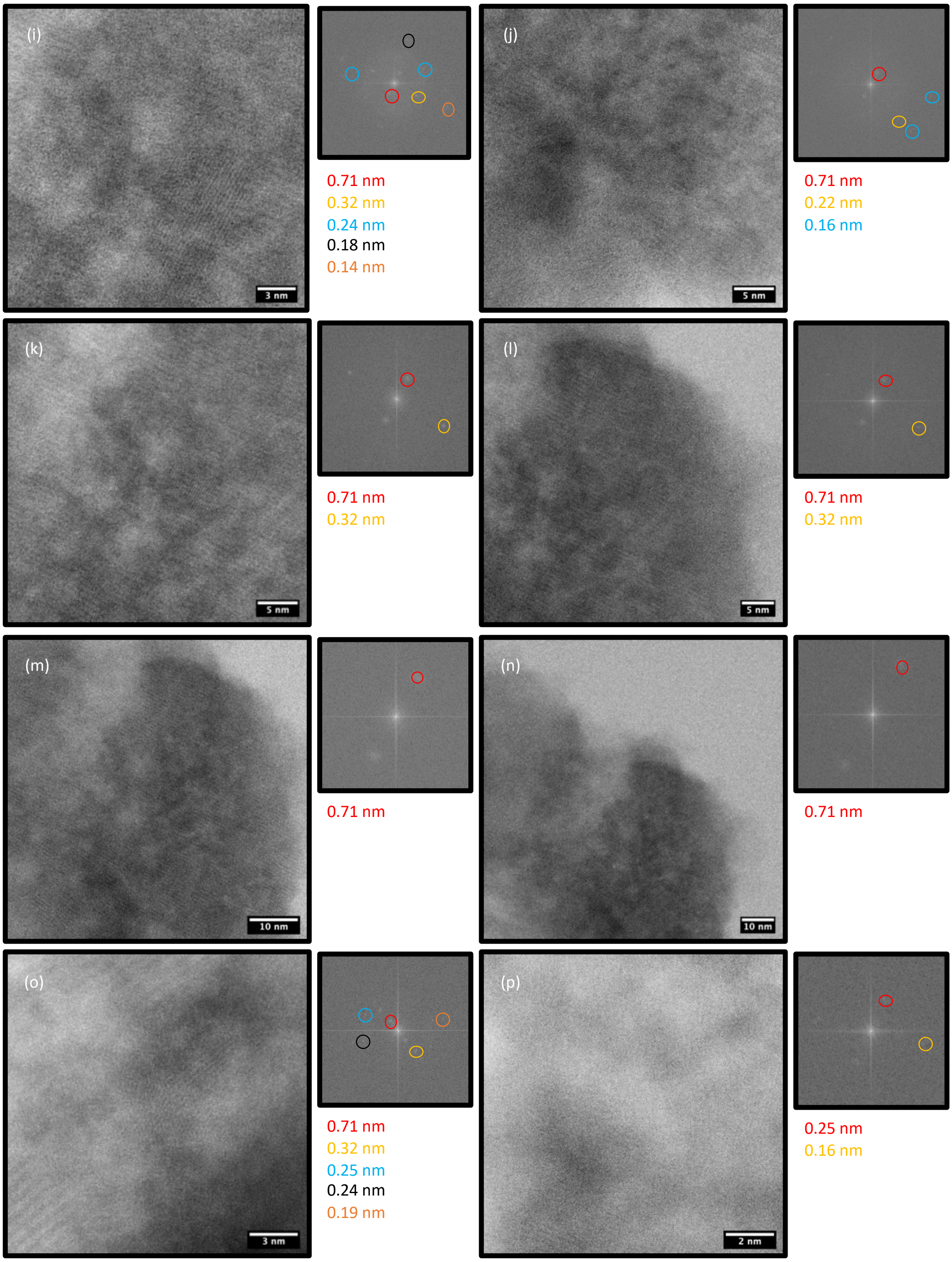

Supplementary Figure 9 Bright field (a-n) and high-angle annular dark field (o-p) HR-STEM images of Fe-BTC. Insets show the Fourier transform of the corresponding micrograph; reciprocal space features correspond to periodic features in the real space image. Coloured circles are used to highlight reciprocal space features and colour-coded distances are given below the Fourier transform.

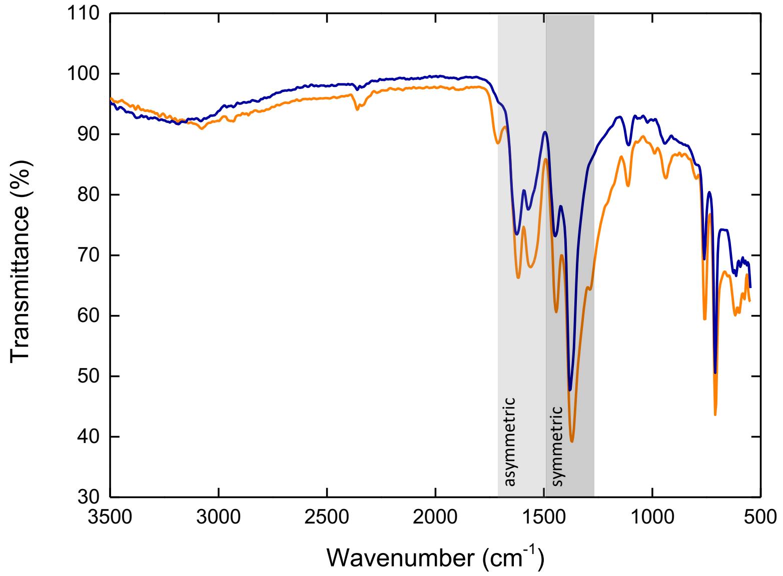

Supplementary Figure 10 FT-IR spectra from Fe-BTC (orange) and MIL-100 (blue). The asymmetric (light grey) and symmetric (dark grey) carboxylate stretching regions are highlighted. Both spectra contain three small bands, at 1,111, 1,713 and 2,361 cm^{-1}, arising from minor impurities of unreacted linker.

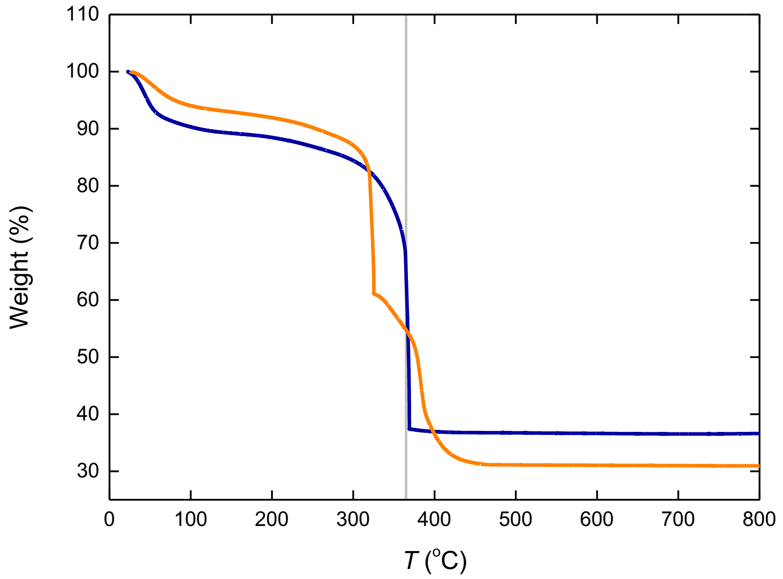

Supplementary Figure 11 TGA analysis, under air, of Fe-BTC (orange) and MIL-100 (blue). Grey line at 365°C highlights the decomposition of MIL-100.

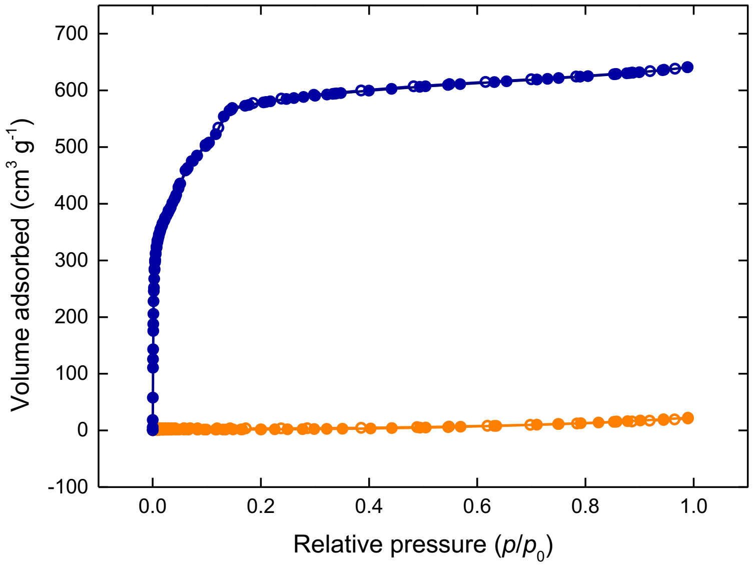

Supplementary Figure 12 Nitrogen adsorption (closed circle) and desorption (open circle) isotherms for MIL-100 (blue) and Fe-BTC (orange).

Supplementary Table 3 BET surface areas of Fe-BTC and MIL-100.

	BET Surface area (m^2 g^{-1})	Maximal uptake (cm^3 g^{-1})	Total pore volume (cm^3 g^{-1})
Fe-BTC	6.17(3)	22.4	0.0356
MIL-100	2240 (18)	641.0	0.991

Supplementary Table 4 Elemental analysis of MIL-100 and Fe-BTC and theoretical values calculated from the empirical formula for MIL-100, $Fe_3O(F)(H_2O)_2[(C_6H_3)(CO_2)_3]_2$.

	MIL-100	Fe-BTC	MIL-100 calculated
C wt.%	31.2	33.6	33.1
H wt.%	2.4	2.41	1.5
N wt.%	0.24	0.57	0

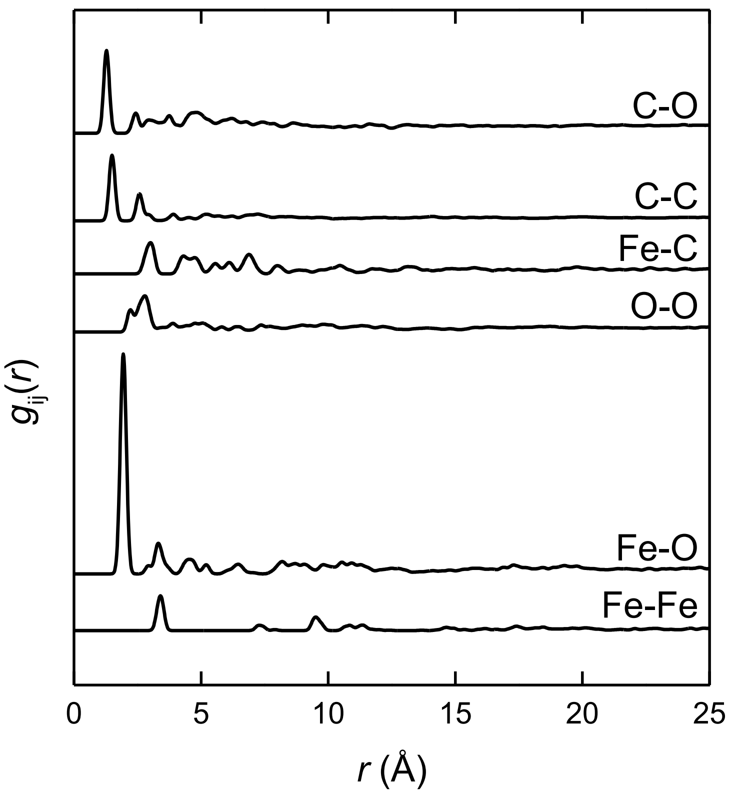

Supplementary Figure 13 Calculated partial pair distribution functions for MIL-100.

Supplementary Table 5 Calculated properties of the three amorphous model types (SRO, MIX and MRO), where each reported value represents the average over the five independent packings and standard deviations in parenthesis. Skeletal densities are calculated by subtracting the volume accessible to a probe radius of 1.3 Å (kinetic diameter of He) from the total volume of the system.

	SRO	MIX	MRO
% Trimer	100	50	0
% Tetrahedra	0	50	100
Final polymerisation (%)	92.1 (1)	92.2 (0.5)	91.5 (0.3)
Number of unreacted sites	189 (24)	187 (11)	205 (6)
Accessible surface area 1.82 Å probe (m^2 g^{-1})	0	0	724 (77)
Non-accessible surface area 1.82 Å probe (m^2 g^{-1})	243 (36)	392 (36)	78.4 (28)
Total surface area 1.82 Å probe (m^2 g^{-1})	243 (36)	392 (36)	803 (51)
Skeletal density (g cm^{-3})	1.57 (0.01)	1.51 (0.01)	1.45 (0.01)
D_i (Å)[a]	8.03 (0.90)	9.15 (0.55)	14.96 (1.67)
D_f (Å)[b]	2.94 (0.11)	3.19 (0.10)	4.92 (0.66)
D_{if} (Å)[c]	7.62 (1.08)	8.18 (0.65)	13.43 (2.37)

[a] Diameter of the largest sphere that can be created in the free volume of the model, [b] diameter of the largest sphere that can percolate through the model (corresponds to the critical window size value), [c] diameter of the largest sphere accessible along the path of D_f.

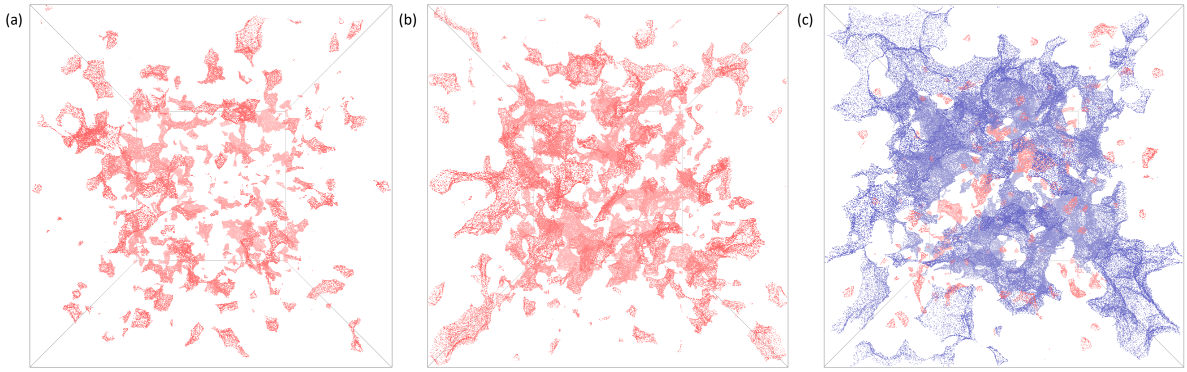

Supplementary Figure 14 The accessible (blue) and non-accessible (red) surface area to a nitrogen probe (3.64 Å diameter) for representative examples of the (a) SRO, (b) MIX and (c) MRO models.

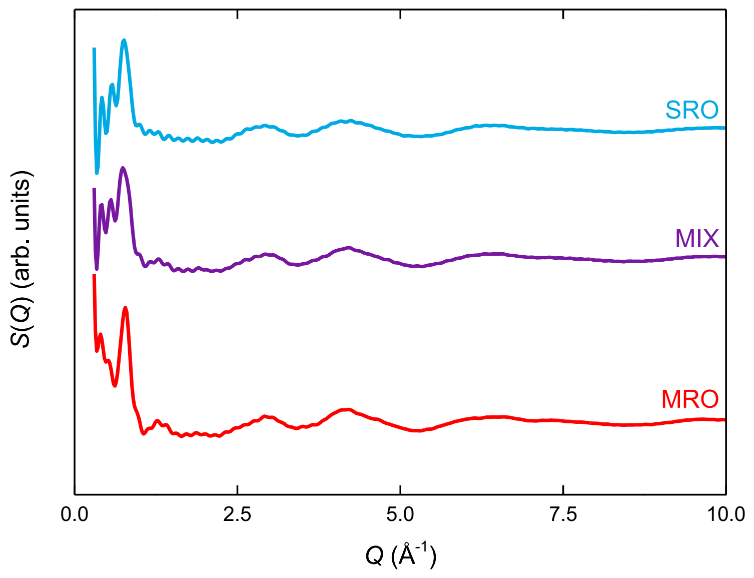

Supplementary Figure 15 Calculated X-ray structure factors for the three model types, curves offset for clarity.

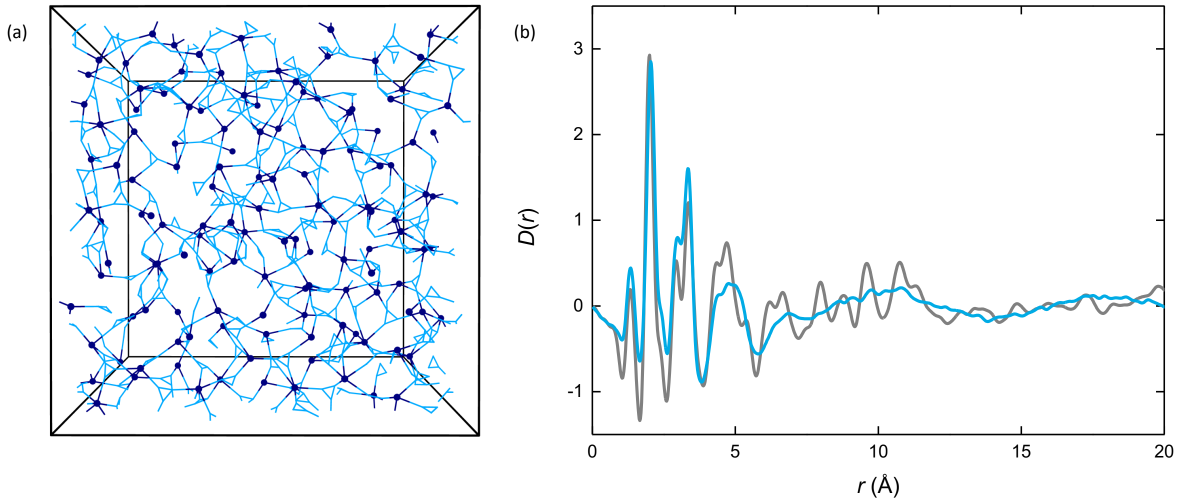

Supplementary Figure 16 (a) Schematic of the SRO model, key shown in Fig. 4. (b) Comparison between the experimental Fe-BTC (grey) and the calculated SRO model (blue) pair distribution functions.

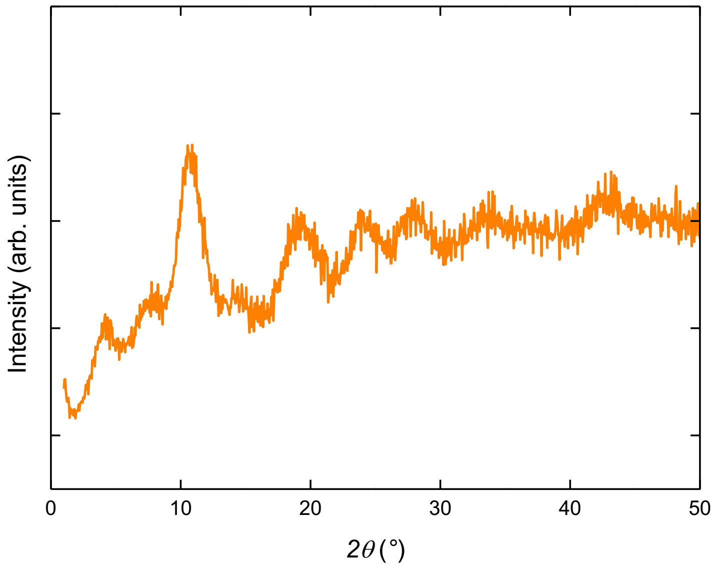

Supplementary Figure 17 Powder X-ray diffraction data from deuterated Fe-BTC.

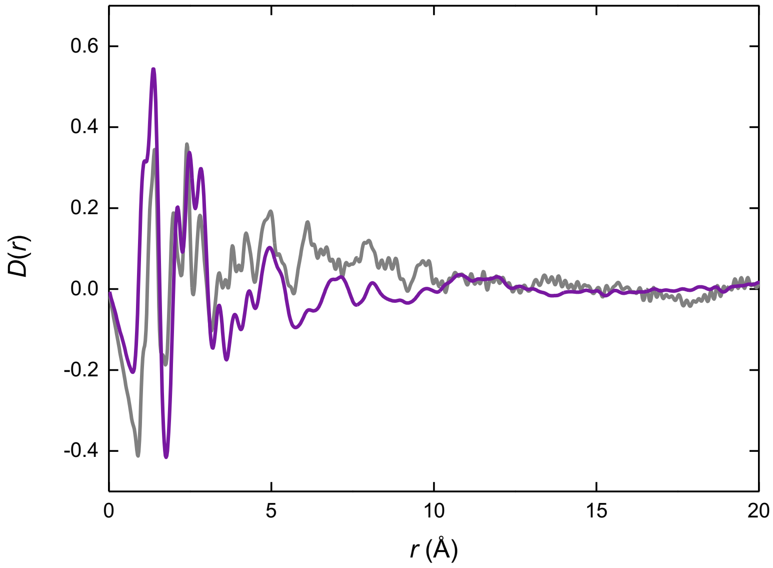

Supplementary Figure 18 Comparison between the neutron pair distribution functions for Fe-BTC (grey) and the calculated data from the MIX model (purple).

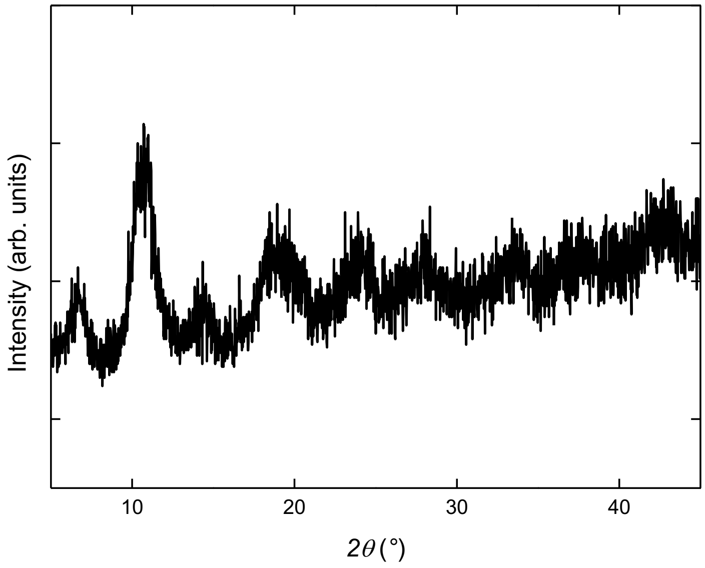

Supplementary Figure 19 Powder X-ray diffraction data for Basolite® F300.

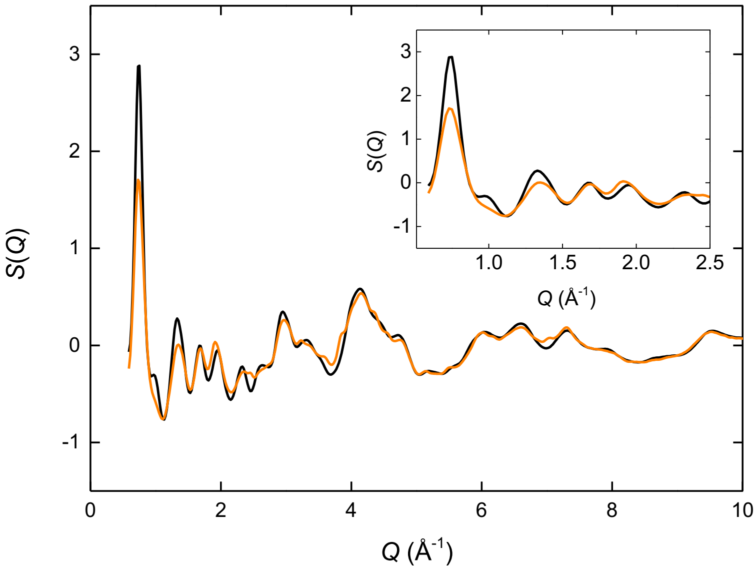

Supplementary Figure 20 X-ray structure factors of Fe-BTC (orange) and Basolite® F300 (black).

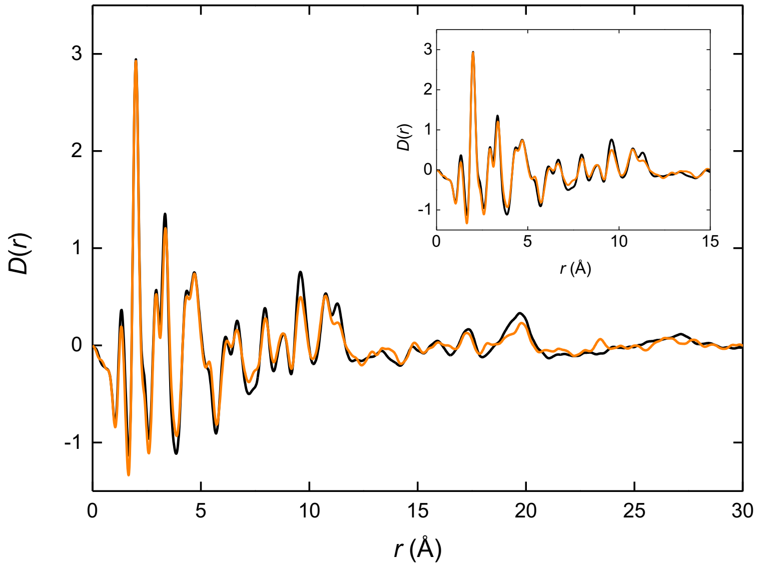

Supplementary Figure 21 X-ray pair distribution functions for Fe-BTC (orange) and Basolite® F300 (black).

Supplementary Methods

Computational methods

Force field validation

The Large-scale Atomic/Molecular Massively Parallel Simulator (LAMMPS) package[1] was used to perform all the energy minimisation and molecular dynamics simulations across the structure generation procedure. All of the structures were described using the extension of the Universal Force Field[2] parametrised for the description of metal–organic frameworks (MOFs), UFF4MOF.[3,4] UFF4MOF has been shown to give a better match with experimental results when used without the implementation of partial charges.[5] Therefore, partial charges were not used in the current models. UFF and UFF4MOF are not currently implemented as a default force field in LAMMPS. To obtain the correct LAMMPS input, the lammps_interface code[5] was used. Firstly, tests were conducted on a simplified fragment (Supplementary Fig. 22), including only one trimer and one 1,3,5-benzenetricarboxylic acid (BTC) linker. However, the comparison between results obtained after geometry optimisation using LAMMPS and using the General Utility Lattice Program (GULP)[6], where UFF4MOF is implemented, led to different values of final energies and geometries. To match final geometry and energies obtained in GULP, we implemented some changes to the LAMMPS input provided by lammps_interface. Firstly, the input files produced by lammps_interface were carefully checked to ensure the correct atom types assignment and that the automatic input generation did not miss bonds, angles, dihedrals or impropers present in the structure. If assigned atom types, bond orders or functional forms were not correct, new corrected coefficients were calculated based on the UFF and UFF4MOF papers.[2-4] Supplementary Figure 22 and Supplementary Table S6 report the UFF4MOF atom types and bond orders, respectively, used in this work.

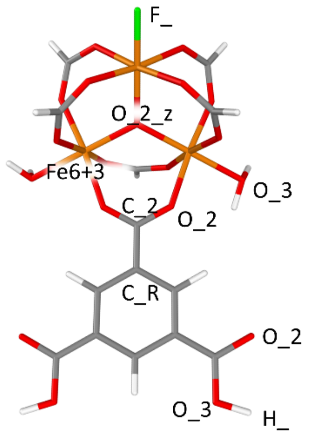

Supplementary Figure 22 UFF4MOF atom types chosen for the cluster used to validate the force field and maintained throughout the rest of the work. Carbon atoms are shown in grey, oxygen in red, iron in orange, fluorine in green and hydrogen in white.

Supplementary Table 6 Selected UFF4MOF bond orders for the atom types involved in the simulated systems.

Atom type1— atom type2	Bond order	Atom type1— atom type2	Bond order
O_2 — Fe6+3	0.5	O_3 — C_2	1
O_2— C_2	1.5	Fe6+3 — F_	1
C_2 — C_R	1	Fe6+3 — O_2_z	0.5
C_R — C_R	1.5	Fe6+3 — O_3	0.5
C_R — H_	1	O_3 — H_	1

The UFF angle potential for 'special geometries', such as trigonal planar or octahedral, is described in UFF in the following way:

$$E_{\theta}(UFF) = \frac{K_{IJK}}{n^2}\left[1 - cos\left(n\theta_{IJK}\right)\right] \qquad (1)$$

where θ is the angle defined by three atoms *I*, *J*, *K*, K_{IJK} is the force constant for the angle bending and *n* is the periodicity of the angle (*n* = 3 for a trigonal planar geometry and *n* = 4 for an octahedral geometry). This can be implemented in LAMMPS using the cosine/periodic angle type, defined as

$$E_{\theta}(LAMMPS) = C\left[1 - B\,(-1)^n cos\left(n\theta_{IJK}\right)\right] \qquad (2)$$

which can be converted into Equation (1) by assigning the three arguments *C*, *B* and *n* the appropriate values. However, the value of E_{ϑ} is multiplied by two when LAMMPS calculates the angle energy of these specific angle types. Therefore, only by dividing the *C* values obtained from lammps_interface for the angle types described with the cosine/periodic angle style (Equation 2) by a factor of two, we could get an exact match between the components of the energy in LAMMPS and in GULP, together with matching geometries. Supplementary Table 7 reports the comparison between the final energies from geometry optimisation with LAMMPS and GULP, while Supplementary Table 8 compares the obtained geometries with the DFT optimised cluster in Gaussian16[7] at the B3LYP-D3/6-31G[8,9] level of theory with an effective core potential on the Fe atoms (B3LYP-D3/LANL2DZ).

Supplementary Table 7 Comparison between energetic terms obtained after optimisation with the UFF4MOF in GULP and the UFF4MOF input adapted for LAMMPS used in this work. Small differences are expected to come from different algorithms used for the optimisation.

Software	Total energy (kcal·mol^{-1})	Two-body contribution (kcal·mol^{-1})	Angle contribution (kcal·mol^{-1})	Dihedral contribution (kcal·mol^{-1})	Improper contribution (kcal·mol^{-1})
LAMMPS	104.26	16.67	86.38	1.22	0.00
GULP	104.27	16.67	86.38	1.22	0.00

Supplementary Table 8 Comparison of distances in the test fragment represented in Supplementary Fig. 22 obtained after UFF4MOF and DFT optimisation. Input files and optimised geometries are reported in https://github.com/Ibechis/FeBTC_models.

Distance	UFF4MOF (LAMMPS)	UFF4MOF (GULP)	DFT (B3LYP-D3/6-31G)
Fe–O_{Carb}	2.05 Å	2.05 Å	1.92-1.96 Å
Fe–O_{Oxo}	1.99 Å	1.99 Å	1.84-1.96 Å
Fe–O_{water}	2.08 Å	2.08 Å	2.02 Å
Fe–F	1.85 Å	1.85 Å	1.77 Å
C–O_{trimer}	1.27 Å	1.27 Å	1.27-1.29 Å
C_{carb}–C_{ring}	1.48 Å	1.48 Å	1.48 Å
C_{ring}–C_{ring}	1.41 Å	1.41 Å	1.40 Å
O–H	0.99 Å	0.99 Å	0.97-0.98 Å
Fe–Fe	3.45 Å	3.45 Å	3.2-3.3

LAMMPS data files and the input file for the fragment minimisation using UFF4MOF can be found at https://github.com/Ibechis/FeBTC_models, together with the input used for the comparison in GULP.

Amorphous model construction

The amorphous structural models for Fe-BTC were generated with the polymerisation algorithm Polymatic,[10] as previously carried out for the amorphous structure of amorphous ZIF-4.[11] This approach gave good results in terms of densities and porosities when compared to experimentally available values for *a*ZIF-4. The workflow followed to obtain the structure is reported in Supplementary Fig. 23.

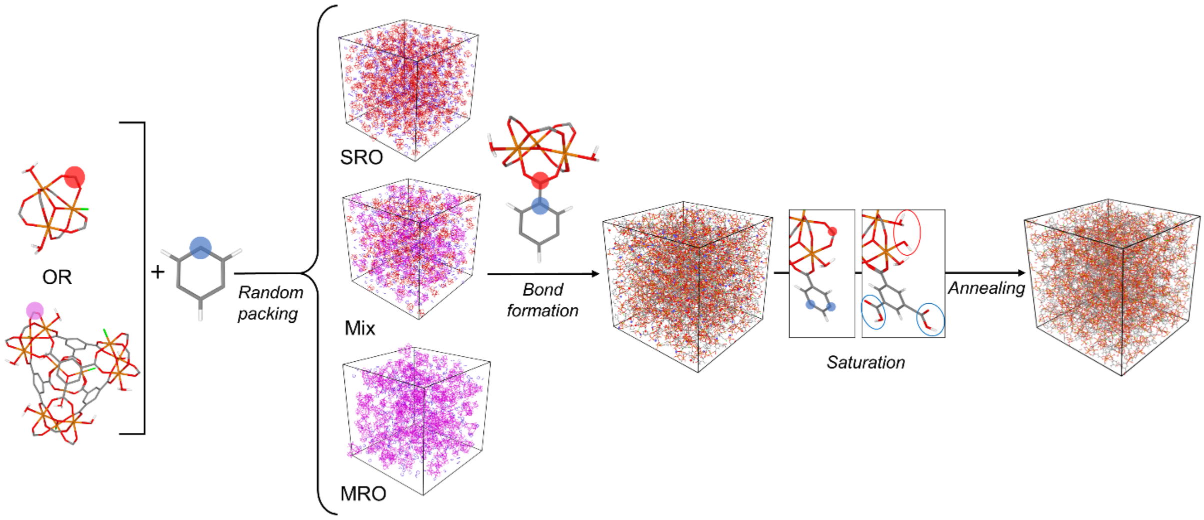

Supplementary Figure 23 Representation of the procedure used to build the SRO, MIX and MRO amorphous models. After having randomly packed the building blocks of the structure in a periodic box, bonds are formed between the defined reactive sites. At the end of the bond formation, unreacted sites are saturated with capping group and the structure is annealed through the 21-step MD protocol (Supplementary Table 10). Carbon atoms are reported in grey, oxygen in red, iron in orange, fluorine in green and hydrogens in white. The circles in the building blocks (blue for the linker, red for the trimer and pink for the tetrahedra) highlight the selected reactive sites. In the random packing, packed trimers are reported in red, linkers in blue and tetrahedra in pink.

Random packing

In order to have a statistical representation of the material, five independent models for each system were generated by randomly packing the inorganic and organic building blocks in a periodic box. Supplementary Figure 23 shows the different building blocks used to account for different degrees of disorder in the final model. In the model that maintains only the short-range order (SRO), 400 trimers and 800 BTC linkers were packed in a periodic box with sides of 80 Å in length (initial density of 0.847 g cm^{-3}). In the model that maintains the medium-range order (MRO), 100 tetrahedra (one tetrahedron includes four trimers and four BTC linkers already connected in the tetrahedra topology) and 400 BTC linkers were packed in a periodic box with sides of 90 Å in length (initial density of 0.595 g cm^{-3}). In the MIX model, 50 tetrahedra (which corresponds to 200 trimers, 50% of the total number of trimers) and 200 free trimers were packed together with 600 BTC linkers in a periodic box with sides of 85 Å in length (initial density of 0.706 g cm^{-3}). All the random packings respect a one-to-two trimer-to-linkers ratio (one trimer is hexacoordinated, while one linker is tricoordinate) and yielded initial models with 18,800 atoms.

Bond creation and polymerisation

In the polymerisation phase, bonds are formed between reactive atoms on opposite building blocks within a cut-off distance of 5 Å. The reactive groups were the carbon in the formate unit coordinated to the Fe atoms in the trimer (red circle in Supplementary Fig. 23) and the aromatic carbon in the BTC linker (blue circle in Supplementary Fig. 23). Intermediate molecular dynamics (MD) steps were performed once every five new bonds formed in the canonical (NVT) or in the isothermal isobaric (NPT) ensembles in an alternating fashion, to allow the structure to adapt and the bond formation to continue, MD steps also help opposite reactive sites to find each other. The NVT MD steps were performed at 1000 K for 10 ps using a timestep of 1 fs. The NPT MD steps were performed at 400 K and 1 bar for 5 ps using a timestep of 1 fs. The Lennard-Jones (LJ) potential was used to model the short-range van der Waals interactions. A cut-off distance for the LJ interactions and the real part of the Ewald summation was set to 15 Å. Constant pressure and temperature were maintained using a Nosé-Hoover thermostat and barostat during MD steps. Opposite fractional charges of 0.5 *e* were added to opposite reactive sites to aid the bond formation. Temperature, duration, artificial charge values and cut-off distances for the polymerisation were chosen in order to obtain the highest possible degree of polymerisation.

Results of an initial benchmark for the polymerisation conditions conducted on a small system (2,350 atoms) are reported in Supplementary Table 9, where the final degree of polymerisation is averaged over three models and standard deviations are reported in parenthesis. We observed that with cut-off values greater than 5 Å, the creation of new bonds sometimes led to distorted structures that caused a failure in the calculation. To avoid the possibility of forming physically unrealistic bonds between building blocks (see Supplementary Fig. 24a) in which one linker is bonded more than once to the same trimer, the 'intra' flag in Polymatic was set equal to five, to check that the building blocks belonging to the newly formed bond were not already connected within five atoms from the reactive

sites. In addition, small modifications of the Polymatic scripts were necessary to handle LAMMPS files described with UFF4MOF, which is a class I force field, as the original scripts are written for class II force fields, like the polymer consistent force field (pcff).[12] These changes mainly include modifications in order to read/write the correct number of arguments for the force field parameters section in the LAMMPS files and modifications in the definition of improper torsions. For an improper torsion involving four atoms *i*, *j*, *k* and *l*, the original Polymatic scripts define the atom *j* as the central atom, while the improper style used by UFF4MOF in LAMMPS defines atom *i* as the central atom. This would lead to errors in the description of the new improper torsions defined after the formation of a new bond, because Polymatic would print out the atoms belonging to the new improper torsions in the wrong order.

Supplementary Table 9 Percentages of polymerisation reached with different polymerisation setups. Tests were done on a small system of 2,350 atoms and averaged over three different models. Standard deviations are reported in parenthesis. Entry 31 (reported in green) is the final polymerisation set-up used in this work.

Set-up number	Cut-off radius (Å)	Additional Charge (*e*)	frequency of NPT MD	Temperature of NVT MD (K)	Polymerisation (%)
1	4	0.0	never	800	66.8 (1.1)
2	4	0.0	never	1000	67.8 (1.9)
3	4	0.0	never	1200	69.4 (0.6)
4	4	0.0	every 10 bonds	800	9.5 (1.4)
5	4	0.0	every 10 bonds	1000	9.3 (0.4)
6	4	0.0	every 10 bonds	1200	8.5 (0.7)
7	4	0.0	every 30 bonds	800	35.6 (8.5)
8	4	0.0	every 30 bonds	1000	38.8 (19.6)
9	4	0.0	every 30 bonds	1200	24.3 (5.0)
10	4	0.3	never	800	77.2 (1.3)
11	4	0.3	never	1000	77.8 (2.3)
12	4	0.3	never	1200	78.7 (1.2)
13	4	0.3	every 10 bonds	800	84.1 (1.4)
14	4	0.3	every 10 bonds	1000	85.2 (1.6)
15	4	0.3	every 10 bonds	1200	84.1 (1.5)
16	4	0.3	every 30 bonds	800	83.1 (2.2)
17	4	0.3	every 30 bonds	1000	84.1 (1.2)

18	4	0.3	every 30 bonds	1200	85.1 (0.8)
19	4	0.5	never	800	83.8 (0.5)
20	4	0.5	never	1000	84.1 (0.7)
21	4	0.5	never	1200	83.3 (2.2)
22	4	0.5	every 10 bonds	800	88.2 (0.9)
23	4	0.5	every 10 bonds	1000	89.8 (1.3)
24	4	0.5	every 10 bonds	1200	89.6 (0.6)
25	4	0.5	every 30 bonds	800	88.5 (1.6)
26	4	0.5	every 30 bonds	1000	88.5 (2.4)
27	4	0.5	every 30 bonds	1200	87.8 (0.2)
28	5	0.3	every 10 bonds	1000	85.1 (1.9)
29	5	0.3	every 10 bonds	1000	89.2 (0.5)
30	5	0.5	every 10 bonds	1000	88.5 (1.6)
31	5	0.5	every 10 bonds	1000	91.6 (2.5)

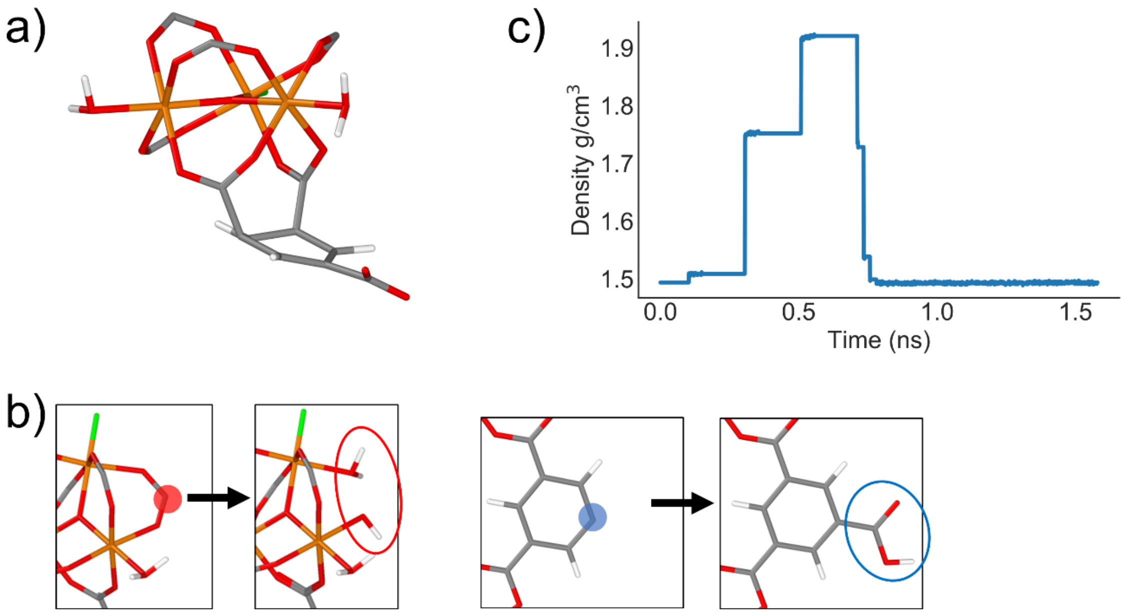

Supplementary Figure 24 (a) An example of an unrealistic bond between building blocks that can be formed in Polymatic if the intra flag is not used. (b) Capping groups used in the saturation step for unreacted trimers (red circle) or unreacted linkers (blue circle). (c) Plot of the system density during the 21-step annealing process. Carbon atoms are reported in grey, iron atoms in orange, fluorine in green and hydrogens in white.

Saturation and introduction of defects in the system

After polymerisation, all the additional charges used to help the polymerisation are removed and unreacted sites are saturated with capping groups. The chosen saturation groups for the unreacted sites are shown in Supplementary Fig. 24b: unreacted formate groups on trimers were substituted with a water molecule and a hydroxide group, while unreacted aromatic carbons on the linker were completed with the carboxylic acid functionality. In this way, the saturation is just an overall addition of two water molecules for each missed bond. As the system is treated without the implementation of partial charges, after the saturation the overall charge of the system is zero.

Several studies have been performed from both the computational and experimental points of view to investigate the nature of missing linker and missing node defects in different MOFs.[13–20] To the best of our knowledge there are no studies investigating the nature of the capping group in the case of a missing-linker defect in MIL-100(Fe). Several studies suggested the presence of additional Brønsted acid sites in Fe-BTC when compared to MIL-100.[21,22] The choice of using a water molecule and a hydroxide group to saturate the two Fe atoms resulting from a missing BTC linker (Supplementary Fig. 24b) was inspired by the formation of similar missing linker defects in UiO-66 in presence of water molecules.[13]

21-step annealing towards a realistic structural model

The polymerised and saturated structures were then annealed through a 21-step MD equilibration, an established protocol that has been developed to obtain realistic structures of microporous polymers.[10] The goal of the annealing process is to produce a physically sensible structure, as the initial random packing and polymerisation are performed at a lower density to help bond formation. A final temperature value (T_{Final}) of 300 K, a maximum temperature value (T_{Max}) of 1000 K, a final pressure value (P_{Final}) of 1 bar and a maximum pressure value (P_{Max}) of 5 x 10^4 bar were used for the annealing process. Supplementary Table 10 reports all the conditions for the 21 steps of the annealing and Supplementary Figure 24c shows the change in pressure as a function of simulation time during annealing. The whole procedure was performed in the gas phase, as the goal is not to exactly reproduce the synthesis procedure and conditions, but to build a model that represents the material obtained experimentally.

Supplementary Table 10 Conditions of the 21 molecular dynamics steps performed during the annealing procedure. Taken from Ref 10.

Step	Ensemble	Conditions	Duration (ps)
1	NVT	T_{Max}	50
2	NVT	T_{Final}	50
3	NPT	T_{Final}, 0.02 (P_{Max})	50
4	NVT	T_{Max}	50
5	NVT	T_{Final}	100
6	NPT	T_{Final}, 0.6 (P_{Max})	50
7	NVT	T_{Max}	50
8	NVT	T_{Final}	100
9	NPT	T_{Final}, P_{Max}	50

10	NVT	T_{Max}	50
11	NVT	T_{Final}	100
12	NPT	T_{Final}, 0.5 (P_{Max})	5
13	NVT	T_{Max}	5
14	NVT	T_{Final}	10
15	NPT	$T_{Final,}$ 0.1 (P_{Max})	5
16	NVT	T_{Max}	5
17	NVT	T_{Final}	10
18	NPT	T_{Final}, 0.01 (P_{Max})	5
19	NVT	T_{Max}	5
20	NVT	T_{Final}	10
21	NPT	T_{Final}, P_{Final}	800

Optimisation of the MIL-100(Fe) crystal with UFF4MOF

The crystal structure of MIL-100(Fe) was optimised with UFF4MOF in LAMMPS, in order to compare it with both the calculated and experimental amorphous phases. The asymmetric unit was obtained from the CoRE MOF database[23] (refcode: CIGXIA) and then modified by adding two water molecules and one fluorine atom in the sixth coordination position of the Fe atoms in each trimer, to make the building blocks comparable to the ones used for the amorphous models. These modifications were done using Material Studio.[24] The final optimised unit cell contains 12784 atoms and has a lattice parameter of 73.655 Å (experimentally reported lattice parameter is 73.340 Å). Comparison between the calculated and experimental PDF is reported in Supplementary Fig. 25.

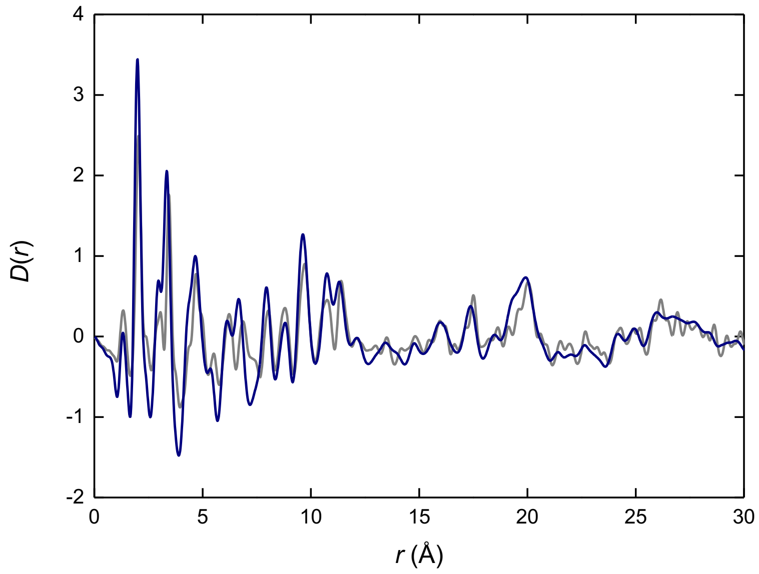

Supplementary Figure 25 Comparison between the experimental MIL-100 PDF (blue) and the calculated PDF from the optimised MIL-100 structure (grey).

Porosity evaluation of amorphous and crystalline models

The calculated porosity of the amorphous models were compared to that of the crystalline MIL-100 model by averaging geometric measures of porosity (calculated using Zeo++[25]) over the final structure obtained from the annealing process of all five models of each system. During the porosity analysis, the structure is kept rigid, therefore the flexibility of the system and the potential swelling induced by adsorption are not considered. The accessible and non-accessible geometric surface areas were calculated with a probe diameter of 3.64 Å (kinetic diameter of N_2).[26] Surface areas were calculated using 7000 Monte Carlo (MC) samples per atom. Pore size distributions were calculated using 70000 MC samples per cell. For the visual pore size distribution 500000 MC samples were used and the results were visualised using OVITO.[27] All the calculations were performed using the high accuracy flag. Final results for all the systems are reported in Supplementary Table 5. Pore size distributions calculated with a probe radius of 0.1 Å are reported in Supplementary Figure 26. The crystal model shows a pore size distribution with peaks at 20-22 Å and 28 Å. These values are slightly lower than the experimentally reported pore values of 25 and 29 Å and they can be justified by the addition of the two water molecules and the fluorine groups in the model.[28] The visual pore size distributions for all the systems calculated with a probe radius of 1.2 Å are reported in Supplementary Figure 27.

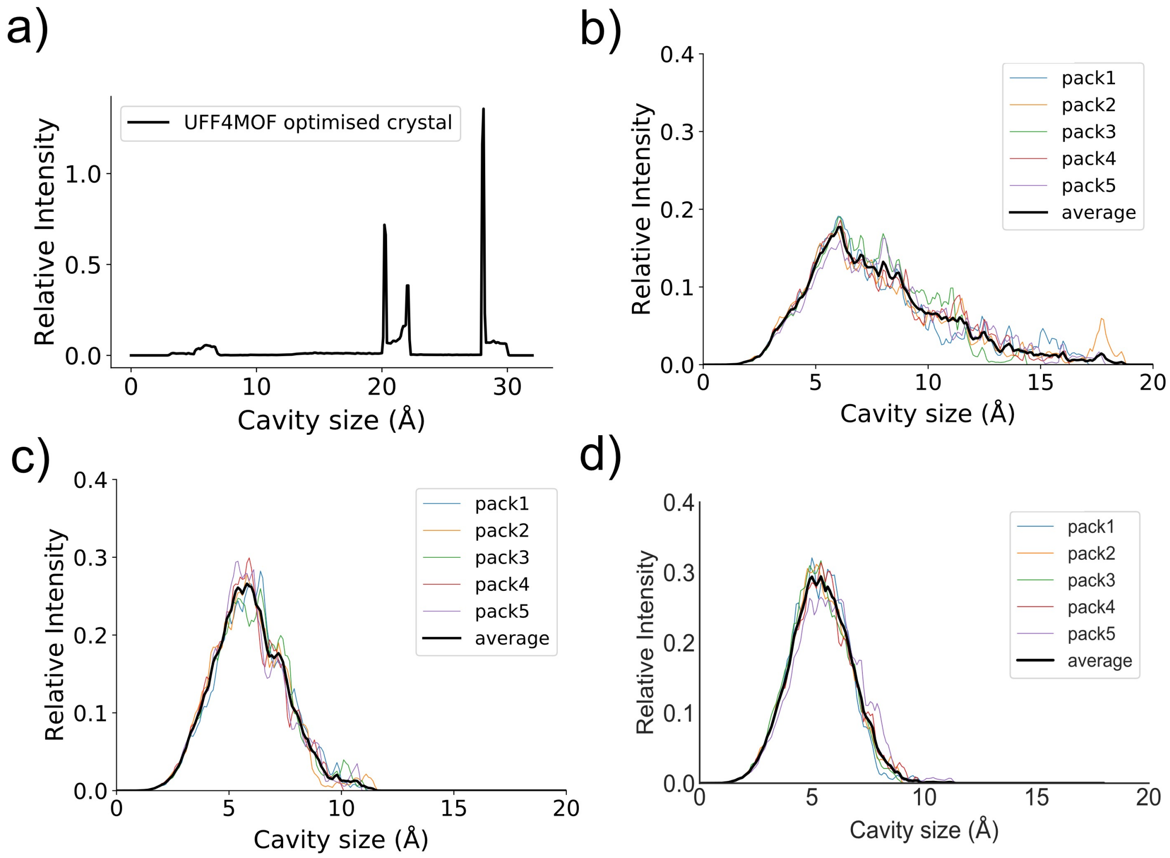

Supplementary Figure 26 Pore size distributions of a) the crystalline MIL-100 model b) the MRO amorphous models c) the MIX amorphous models and d) the SRO amorphous models calculated with a probe radius of 0.1 Å (the average over the five packings is reported in black).

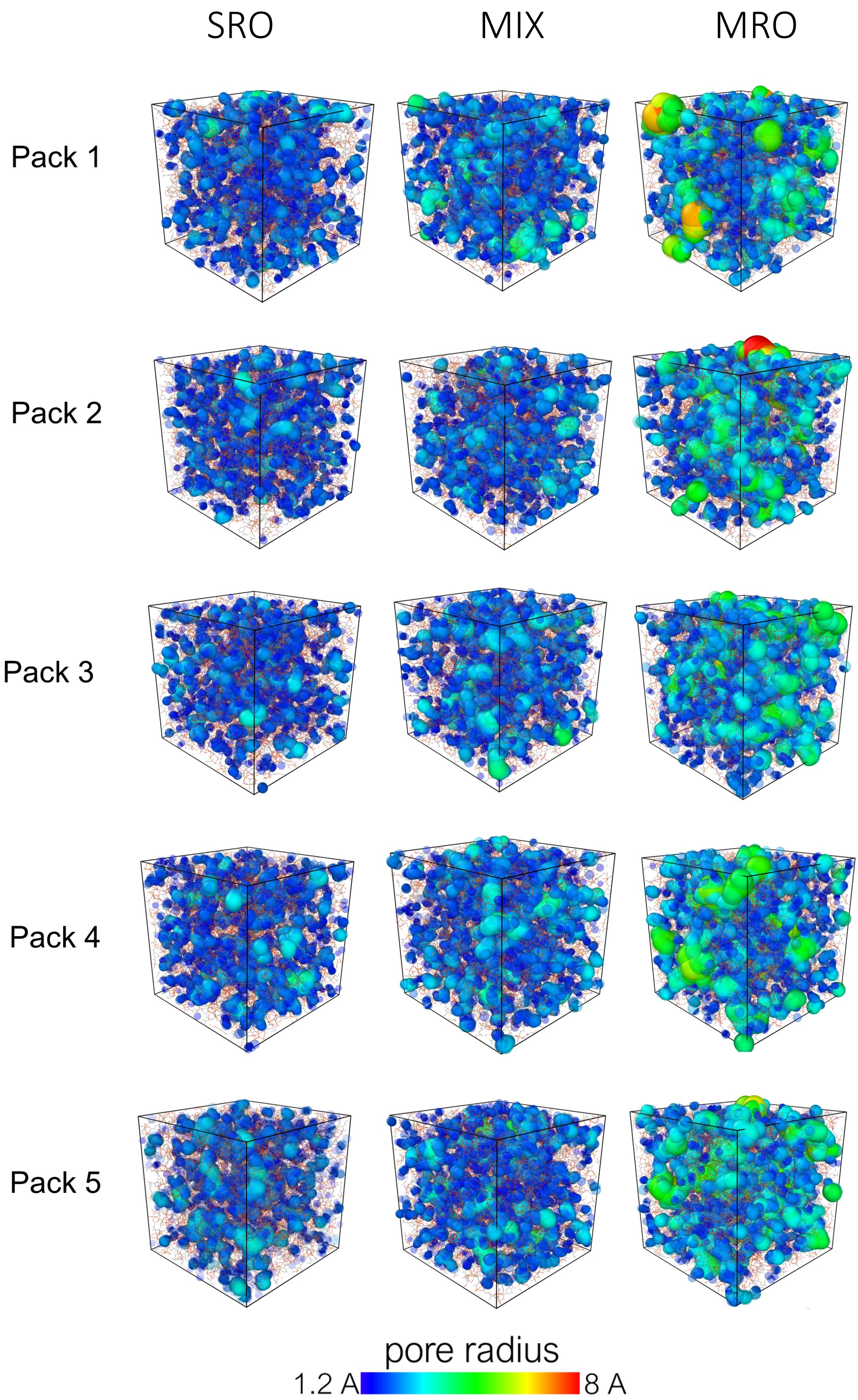

Supplementary Figure 27 Visual pore size distribution for all the amorphous models, calculated with a 1.2 Å probe radius.

Supplementary References

(1) Plimpton, S. Fast Parallel Algorithms for Short-Range Molecular Dynamics. *J. Comput. Phys.* **1995**, *117*, 1–19.

(2) Rappe, A. K.; Casewit, C. J.; Colwell, K. S.; Goddard, W. A.; Skiff, W. M. UFF, a Full Periodic Table Force Field for Molecular Mechanics and Molecular Dynamics Simulations. *J. Am. Chem. Soc.* **1992**, *114*, 10024–10035.

(3) Addicoat, M. A.; Vankova, N.; Akter, I. F.; Heine, T. Extension of the Universal Force Field to Metal−Organic Frameworks. *J. Chem. Theory Comput.* **2014**, *10*, 880−891.

(4) Coupry, D. E.; Addicoat, M. A.; Heine, T. Extension of the Universal Force Field for Metal−Organic Frameworks. *J. Chem. Theory Comput.* **2016**, *12*, 5215−5225.

(5) Boyd, P. G.; Moosavi, S. M.; Witman, M.; Smit, B. Force-Field Prediction of Materials Properties in Metal-Organic Frameworks. *J. Phys. Chem. Lett.* **2017**, *8*, 357–363.

(6) Gale, J. D.; Rohl, A. L. The General Utility Lattice Program (GULP). *Mol. Simul.* **2003**, *29*, 291–341.

(7) Gaussian 16, Revision C.01, Frisch, M. J.; Trucks, G. W.; Schlegel, H. B.; Scuseria, G. E.; Robb, M. A.; Cheeseman, J. R.; Scalmani, G.; Barone, V.; Petersson, G. A.; Nakatsuji, H.; Li, X.; Caricato, M.; Marenich, A. V.; Bloino, J.; Janesko, B. G.; Gomperts, R.; Mennucci, B.; Hratchian, H. P.; Ortiz, J. V.; Izmaylov, A. F.; Sonnenberg, J. L.; Williams-Young, D.; Ding, F.; Lipparini, F.; Egidi, F.; Goings, J.; Peng, B.; Petrone, A.; Henderson, T.; Ranasinghe, D.; Zakrzewski, V. G.; Gao, J.; Rega, N.; Zheng, G.; Liang, W.; Hada, M.; Ehara, M.; Toyota, K.; Fukuda, R.; Hasegawa, J.; Ishida, M.; Nakajima, T.; Honda, Y.; Kitao, O.; Nakai, H.; Vreven, T.; Throssell, K.; Montgomery, J. A., Jr.; Peralta, J. E.; Ogliaro, F.; Bearpark, M. J.; Heyd, J. J.; Brothers, E. N.; Kudin, K. N.; Staroverov, V. N.; Keith, T. A.; Kobayashi, R.; Normand, J.; Raghavachari, K.; Rendell, A. P.; Burant, J. C.; Iyengar, S. S.; Tomasi, J.; Cossi, M.; Millam, J. M.; Klene, M.; Adamo, C.; Cammi, R.; Ochterski, J. W.; Martin, R. L.; Morokuma, K.; Farkas, O.; Foresman, J. B.; Fox, D. J. Gaussian, Inc., Wallingford CT, 2016.

(8) Stephens, P. J.; Devlin, F. J.; Chabalowski, C. F.; Frisch, M. J. Ab Initio Calculation of Vibrational Absorption and Circular Dichroism Spectra Using Density Functional Force Fields. *Phys. Chem.* **1994**, *98*, 11623–11627.

(9) Grimme, S.; Ehrlich, S.; Goerigk, L. Effect of the Damping Function in Dispersion Corrected Density Functional Theory. *J. Comput. Chem.* **2011**, *32*, 1456–1465.

(10) Abbott, L. J.; Hart, K. E.; Colina, C. M. Polymatic: A Generalized Simulated Polymerization Algorithm for Amorphous Polymers. *Theor. Chem. Acc.* **2013**, *132*, 1334.

(11) Thornton, A. W.; Jelfs, K. E.; Konstas, K.; Doherty, C. M.; Hill, A. J.; Cheetham, A. K.; Bennett, T. D. Porosity in Metal–Organic Framework Glasses. *Chem. Commun.* **2016**, *52*, 3750–3753.

(12) Sun, H. Force Field for Computation of Conformational Energies, Structures, and Vibrational Frequencies of Aromatic Polyesters. *J. Comput. Chem.* **1994**, *15*, 752–768.

(13) Ling, S.; Slater, B. Dynamic Acidity in Defective UiO-66. *Chem. Sci.* **2016**, *7*, 4706.

(14) Zhang, C.; Han, C.; Sholl, D. S.; Schmidt, J. R. Computational Characterization of Defects in Metal−Organic Frameworks: Spontaneous and Water-Induced Point Defects in ZIF-8. *J. Phys. Chem. Lett.* **2016**, *7*, 459−464.

(15) Trickett, C. A.; Gagnon, K. J.; Lee, S.; Gándara, F.; Bürgi, H. B.; Yaghi, O. M. Definitive Molecular Level Characterization of Defects in UiO-66 Crystals. *Angew. Chemie - Int. Ed.* **2015**, *54*, 11162–11167.

(16) Svane, K. L.; Bristow, J. K.; Gale, J. D.; Walsh, A. Vacancy Defect Configurations in the Metal-Organic Framework UiO-66: Energetics and Electronic Structure. *J. Mater. Chem. A* **2018**, *6*, 8507–8513.

(17) Liu, L.; Chen, Z.; Wang, J.; Zhang, D.; Zhu, Y.; Ling, S.; Huang, K. W.; Belmabkhout, Y.; Adil, K.; Zhang, Y.; et al. Imaging Defects and Their Evolution in a Metal–Organic Framework at Sub-Unit-Cell Resolution. *Nat. Chem.* **2019**, *11*, 622–628.

(18) Vandichel, M.; Hajek, J.; Ghysels, A.; De Vos, A.; Waroquier, M.; Van Speybroeck, V. Water Coordination and Dehydration Processes in Defective UiO-66 Type Metal Organic Frameworks. *CrystEngComm* **2016**, *18*, 7056–7069.

(19) Øien, S.; Wragg, D.; Reinsch, H.; Svelle, S.; Bordiga, S.; Lamberti, C.; Lillerud, K. P. Detailed Structure Analysis of Atomic Positions and Defects in Zirconium Metal−Organic Frameworks. *Cryst. Growth Des.* **2014**, *14*, 5370−5372.

(20) Bristow, J. K.; Svane, K. L.; Tiana, D.; Skelton, J. M.; Gale, J. D.; Walsh, A. Free Energy of Ligand Removal in the Metal−Organic Framework UiO-66. *J. Phys. Chem. C* **2016**, *120*, 9276−9281.

(21) Dhakshinamoorthy, A.; Alvaro, M.; Horcajada, P.; Gibson, E.; Vishnuvarthan, M.; Vimont, A.; Grenè, J.-M.; Serre, C.; Daturi, M.; Garcia, H. Comparison of Porous Iron Trimesates Basolite F300 and MIL-100(Fe) As Heterogeneous Catalysts for Lewis Acid and Oxidation Reactions: Roles of Structural Defects and Stability. *ACS Catal.* **2012**, *2*, 2060−2065.

(22) Sciortino, L.; Alessi, A.; Messina, F.; Buscarino, G.; Gelardi, F. M. Structure of the FeBTC Metal−Organic Framework: A Model Based on the Local Environment Study. *J. Photochem. Photobiol. A Chem.* **2015**, *119*, 7826−7830.

(23) Nazarian, D.; Camp, J. S.; Chung, Y. G.; Snurr, R. Q.; Sholl, D. S. Large-Scale Refinement of Metal−Organic Framework Structures Using Density Functional Theory. *Chem. Mater.* **2017**, *29*, 2521−2528.

(24) Accelrys Software Inc. Material Studio. San Diego 2007.

(25) Willems, T. F.; Rycroft, C. H.; Kazi, M.; Meza, J. C.; Haranczyk, M. Algorithms and Tools for High-Throughput Geometry-Based Analysis of Crystalline Porous Materials. *Microporous Mesoporous Mater.* **2012**, *149*, 134–141.

(26) Robeson, L. M. Correlation of Separation Factor versus Permeability for Polymeric Membranes. *J. Memb. Sci.* **1991**, *62*, 165–185.

(27) Stukowski, A. Visualization and Analysis of Atomistic Simulation Data with OVITO–the Open Visualization Tool. *Model. Simul. Mater. Sci. Eng.* **2010**, *18*, 015012.

(28) Horcajada, P.; Surblé, S.; Serre, C.; Hong, D.-Y.; Seo, Y.-K.; Chang, J.-S.; Grenèche, J.-M.; Margiolaki, I.; Férey, G. Synthesis and Catalytic Properties of MIL-100(Fe), an Iron(III) Carboxylate with Large Pores. *Chem. Commun.* **2007**, *27*, 2820–2822.
